# Supplementary figures and images for: Background optic flow modulates responses of multiple descending interneurons to object motion in locusts
Source: PLoS One. 2025 Dec 29;20(12):e0312637. doi: 10.1371/journal.pone.0312637 (PMC12747386; doi:10.1371/journal.pone.0312637)

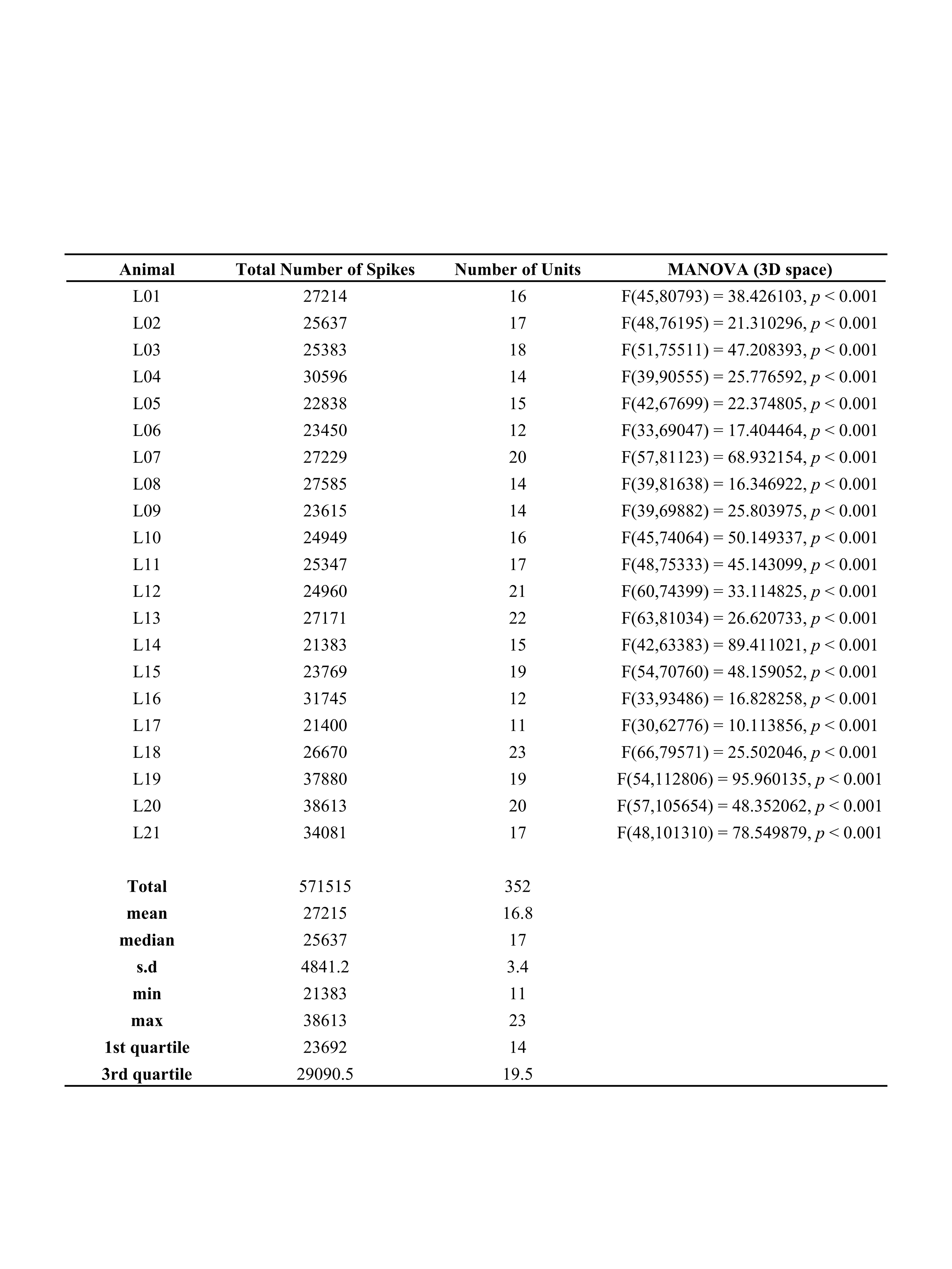

Supplement: S1 Table — On average, 27215 spikes (waveforms) were detected, and 16.8 units were spike-sorted. The MANOVA results show that the spike-sorted units were statistically distinct from each other. (TIF) [file pone.0312637.s001.tif]

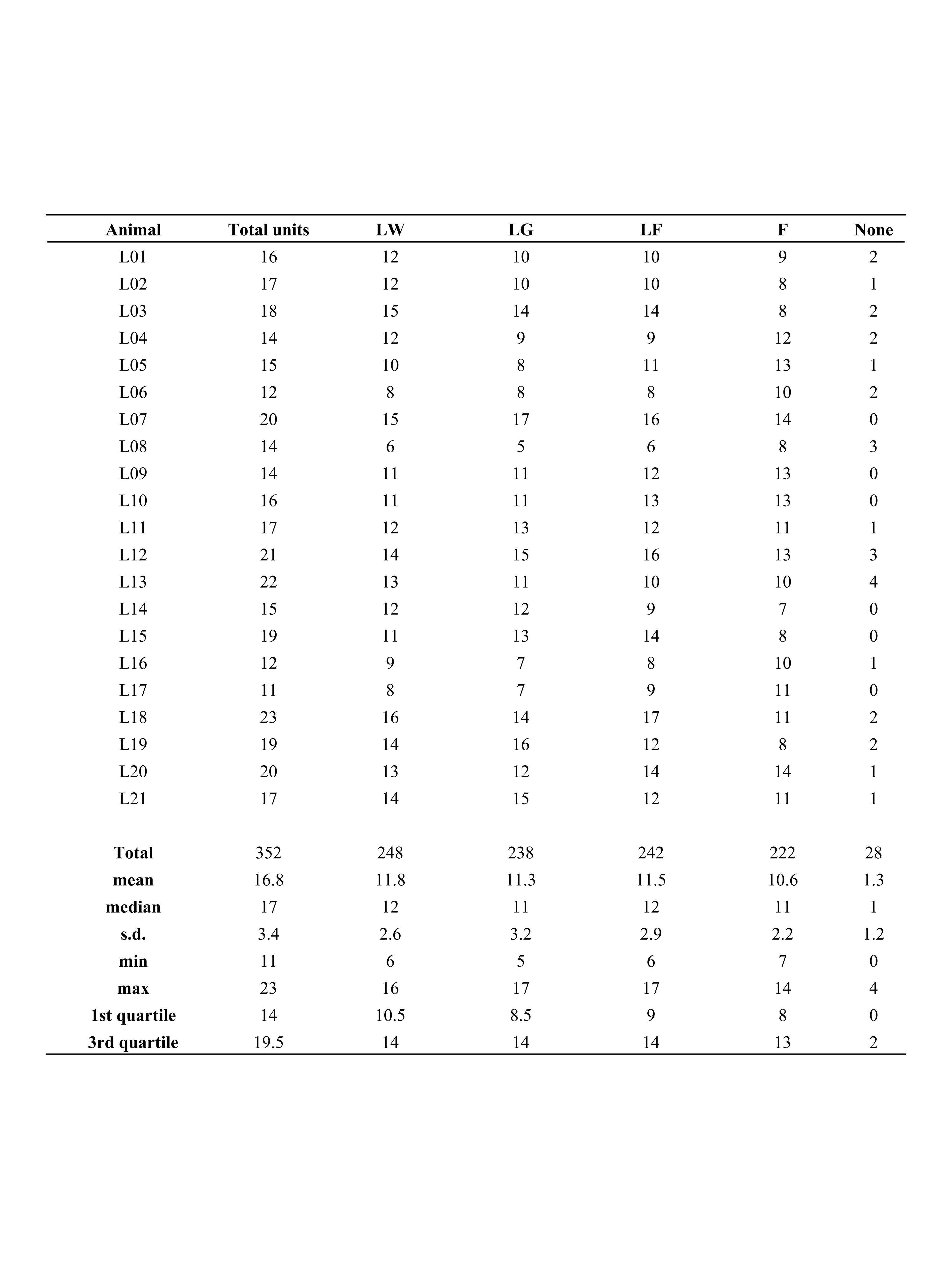

Supplement: S2 Table — LW represents loom against the white background, LG represents loom against the grey background, and LF represents loom against the flow field background. (TIF) [file pone.0312637.s002.tif]

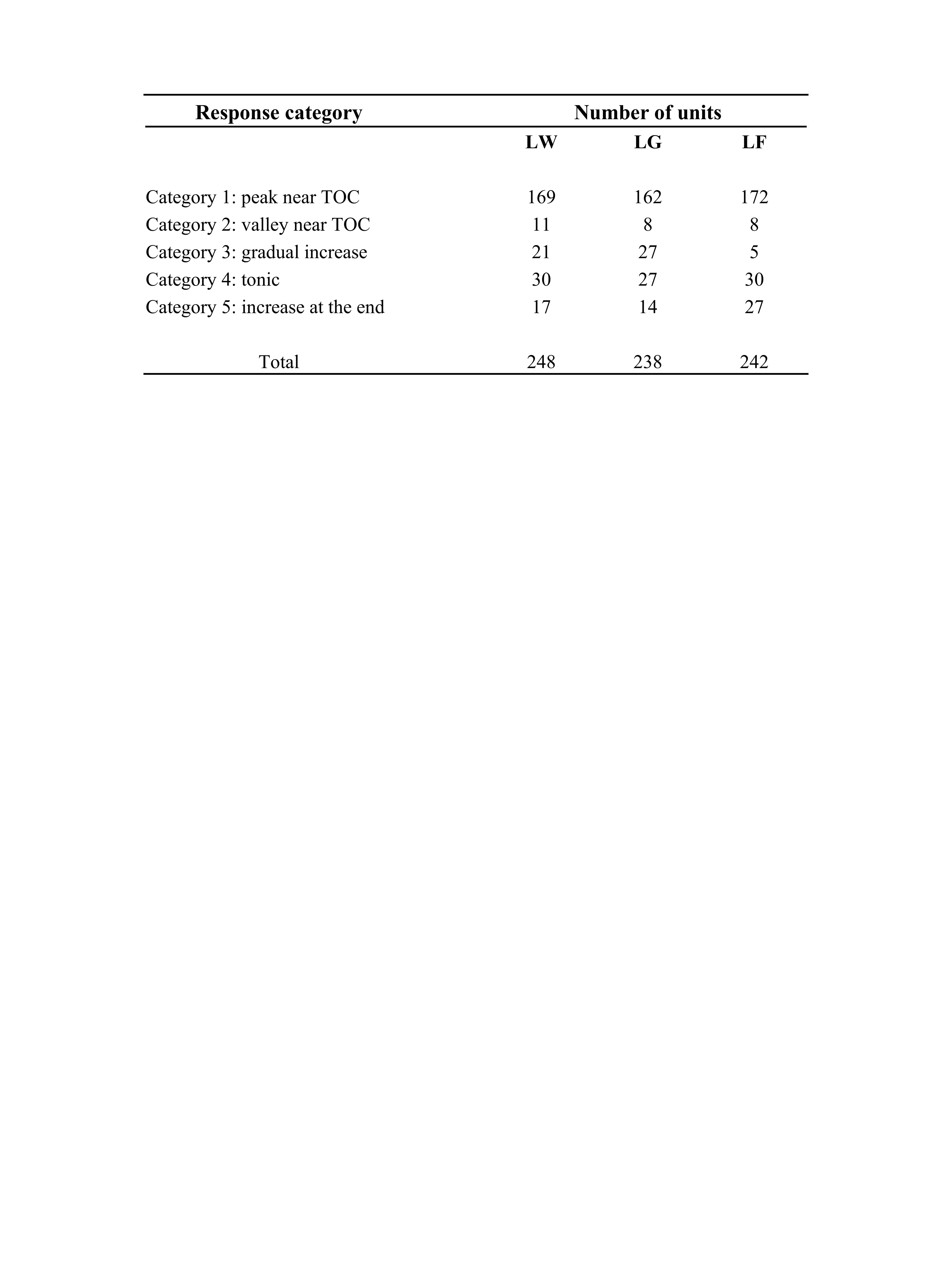

Supplement: S3 Table — The distribution was similar between LW and LG. However, in response to LF, the proportion of Category 3 decreased, while the proportion of Category 5 increased. (TIF) [file pone.0312637.s003.tif]

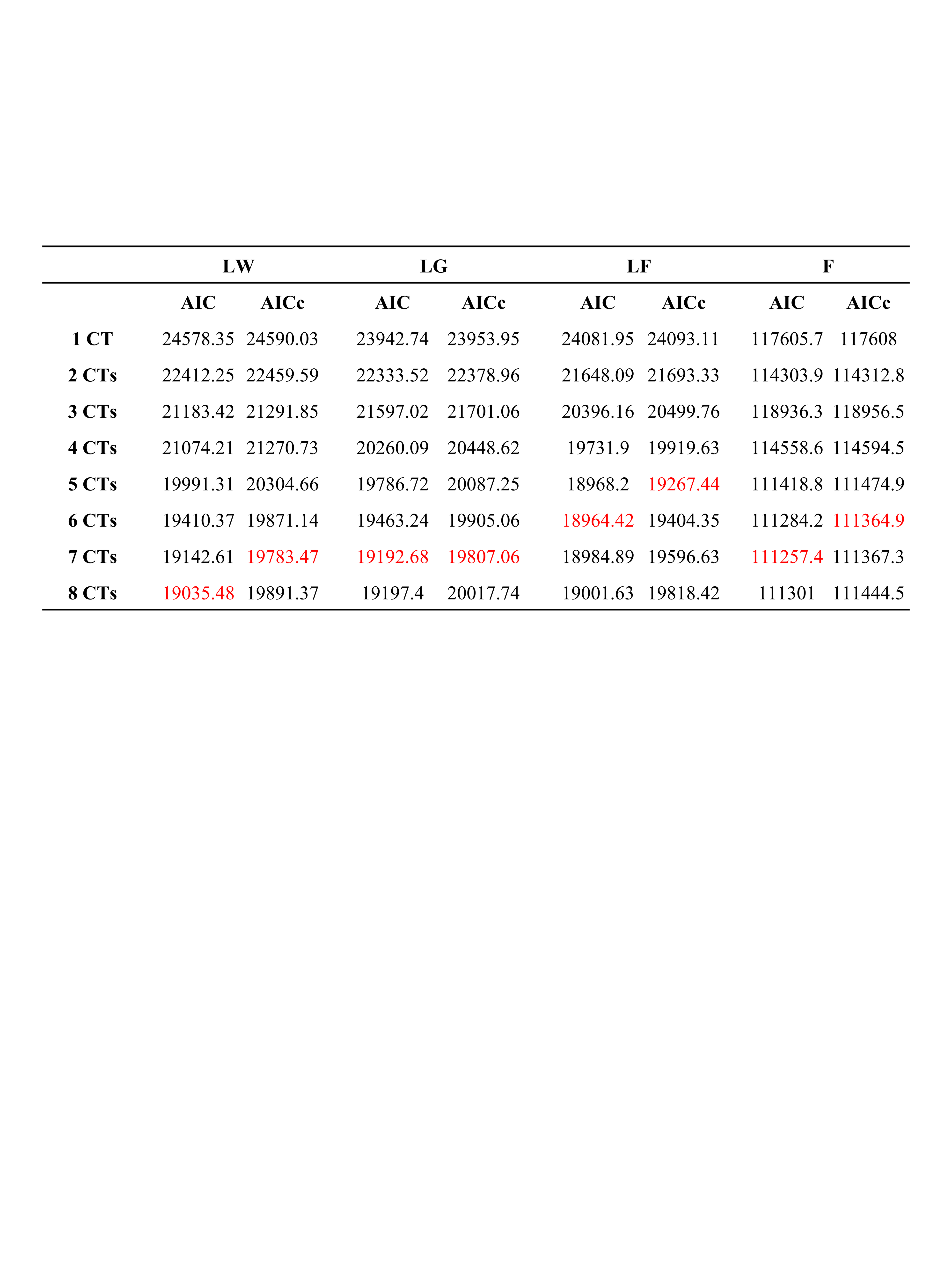

Supplement: S4 Table — For each stimulus type, the DFA model was performed iteratively, starting with 1 common trend (CT). The Akaike information criterion (AIC) and AIC corrected for small sample size (AICc) of each model are shown above. Since AICc can prevent over-fitting, it was used to determine the best-fit approximating model. For looming against both white and white/grey backgrounds, the best models contained 7 common trends. For looming against the flow field background, the best model contained 5 common trends. For flow field only, the best model contained 6 common trends. (TIF) [file pone.0312637.s004.tif]
