# Supplementary material for: Background optic flow modulates responses of multiple descending interneurons to object motion in locusts
Source: PLoS One. 2025 Dec 29;20(12):e0312637. doi: 10.1371/journal.pone.0312637 (PMC12747386; doi:10.1371/journal.pone.0312637)
Supplement: Attachment — Submitted filename: Review.pdf [file pone.0312637.s005.pdf]

In the manuscript 'Background optic flow modulates responses of multiple descending interneurons to object motion in locusts', Zhang and Gray conducted multichannel extracellular recordings from descending neurons of *Locusta migratoria*. Many of the recorded neurons were sensitive to a looming stimulus that mimics a sudden approach of a large object, e.g., a predator. The present study explicitly tested how the presence of optic flow cues in the background affects the neural response to a looming stimulus presented in the foreground. Optic flow cues are visual stimuli induced through self-motion and therefore omnipresent when an insect flies. In other words, testing the neural response to a looming stimulus in the presence of optic flow cues in the background represents a more naturalistic stimulus scenario and hence makes the study's rationale highly attractive to a huge neuroscientific community. In addition to minor concerns, I have only a few major concerns.

Unfortunately, the authors presented mostly grouped data, e.g., figure 5, which makes it difficult for the reader to evaluate the results objectively. More specifically, the reader cannot assess how the response characteristics change with different backgrounds within a unit. As all units were stimulated with all four stimuli, the strength of the author's experimental approach is to show changes in the response profile within a unit. To appreciate the authors' findings, it is important to present response profiles of single units in response to the four different visual stimuli.

Another major point is that the recordings had been conducted in tethered, non-flying locusts. This means that the presented optic flow cues were uncoupled with the locusts' state of locomotion (open loop). The authors must at least discuss the study's technical drawback of recording from a stationary insect. I can imagine that a motor efference copy that closes the loop between the insect's flight movement and the optic flow cues in the background would make the neurons more sensitive to stimuli that mismatch with the motor efference copy, i.e., a looming stimulus that approaches faster than predicted from the insect's flight speed.

Major points:

- Line 144-174: At which velocity was the flow field (background) presented? If it was static, it is critical to call it 'flow'. Based on the introduction, I would suspect to present a looming stimulus in the presence of optic flow that mimics a biologically plausible flight speed of the locust.
- Why did the authors not analyze changes in the response profile induced by different backgrounds for the remaining unit categories (category 2-5)?

Minor points:

Line 52: Please also add a citation of Martin Egelhaaf's group who studied the influence of optic flow on flight control for decades.

Line 64: But, see the recent work of Marion Sillies who revealed six types of direction-sensitive neurons in *Drosophila*. (DOI: 10.1126/sciadv.abi7112)

Line 89-91: Please add a sentence about the nature of your used backgrounds. What were you presenting making them 'naturalistic'?

Line 90: Typo: rigidly-tethered

Line 119: Please specify how the tetrode was fabricated. Which electrodes were used to built the tetrode? Did you measure the electrode resistance before each experiment?

Line 139-140: It might be helpful to add the 'orientation °' in Figure 1C. And please increase the size of the schematized locust in the figure.

Line 165-167: Please explain why you decided to present the looming stimulus from below and not from above?

Line 185-197: How large was the time window for spike detection? Maybe you could add a time scale in Figure 2B.

Line 206: Instead of using the term 3D feature space, I prefer using terms like PCA. Especially, when you are using the abbreviation 'PC' in the figure which has not been introduced in the legend.

Line 213: 'not nonresponded' does not make sense

Line 218: 'discriminated units'. Terms like 'single units' or 'spike-sorted units' are more common.

Line 307: A maximal number of 23 recorded units per animal sounds a lot. Also, the mean of 16.8 units per animal is high. I wonder how the entire PCA of one animal looks. In Figure 2, you are only presenting 3 example units that were relatively easily distinguishable by eye. Is this also the case for an entire PCA?

Line 351-366: Category 4 (Tonic response): How does it differ from a non-responsive unit? Is the firing rate in Figure 5 for category 4 units above a background activity (of no visual stimulus)? Unfortunately, I can´t evaluate this based on the figure.

Line 376: How long does it take until the post-TOC response decays for category 5 units? In Figure 5, it is unfortunately clipped.

Line 385-392: Instead of writing that you found significant differences, specify your effect. For example, for the LF condition the units reached their maximal firing rate later but showed a significant shorter rise phase which reflects a more sudden change in the firing rate than in response to looming stimuli presented in absence of a flow field (LW and LG).

Line 399: You should specify the sample size for decay phase in the corresponding subfigure of figure 6.

Line 400: Typo: 'oof'

Line 424-451: Please specify the rationale for the 'common trend' analysis. Can I interpret it as a model-based categorization of different response profiles (instead of the five categories you manually selected beforehand)?

Line 475: Again specify the sample size

Line 522: Typo: 'bbackground'

Line 522-526: Justifications for the used visual stimuli must be additionally mentioned in the method section. These questions I was asking myself the entire manuscript and had to wait for the answers until the discussion.

Line 532: 'number of common trends, representing the types of responses among all responsive units'. This info must appear in the result section about the common trends.

Line 539: Missing punctuation mark.

Line 585: 'In our study'

Line 560-562: In line with this hypothesis, state-dependent central-complex neurons have been recently described in monarch butterflies (doi.org/10.1016/j.cub.2021.11.009).

Figure 2:

- As you are using two different color codes (one for discriminating the signal from each electrode and one for each sorted unit) I needed some time to understand which color stands for what. I suggest keeping the raw Tetrode signal in grayscale and using the color code only for the sorted units.
- Maybe you could rotate the PCA a little so that the distinction between the green and yellow unit becomes clearer. You could also zoom in a little.
- Please color code the ellipses in **C** so that they can be clearly distinguished from the cumulative response.
- Please indicate from your 20 example units which ones were declared as responsive.

Figure 3:

- For a better comparison across animals, I would plot on the y-axis the percentage of responsive neurons from the recorded neurons rather the absolute number.
- Please add individual data points into your boxplots.

Figure 4:

- Please explain all abbreviations used in the figure in the legend (this comment should be considered for all figures)
- Typo: I assume, on the x-axis it should say ‘F’ instead of ‘FF’?

Figure 5:

- Please indicate the number of neurons presented in each category.

Figure 6:

- Please indicate the number of neurons presented in the boxplots (based on the results I suspect N = 135).
- Please divide the subfigure B into four subfigures (B-E). This makes it much more easier to connect the written results directly to the right subfigure. Also rearrange the boxplot figures so that they fit with the order mentioned in the results (‘peak time’, ‘rise phase’, ‘peak firing rate’, ‘decay phase’).
