# Supplementary material for: Background optic flow modulates responses of multiple descending interneurons to object motion in locusts
Source: PLoS One. 2025 Dec 29;20(12):e0312637. doi: 10.1371/journal.pone.0312637 (PMC12747386; doi:10.1371/journal.pone.0312637)
Supplement: Attachment — Submitted filename: Response to reviewers comments.docx [file pone.0312637.s007.docx]

We thank the reviewers for their carefully considered and constructive comments on the manuscript. We have made an effort to incorporate all suggestions and edits and provide a response to each comment below in red text.

**Reviewer #1:**

The paper by Zhang et al. represents yet another contribution to our extensive and growing knowledge related to the response of motion-sensitive descending interneurons in the locust. This well-characterized system has successfully served for many years as a model for the study of visual responses in complex, dynamic environments.

In this report, the authors present a statistically extensive analysis of the multi-unit response to looming stimuli over different visual backgrounds, including a flow-field background. The major finding is that, while background complexity has some effect on the temporal aspects of the multi-unit response, the basic looming response remains intact.

While somewhat difficult to follow due to the nature of the work, I find that, overall, the paper is well-written and the findings are well-presented. Below, I list some minor as well as more major comments (in order of appearance in the text rather than importance):

**1. L 66: Density dependent phase in locusts is much more than a morphological state.**

We have changed this sentence to more accurately state that migratory locusts are polyphenic.

**2. Figure 1: I suggest adding an indication of the direction of motion of the wide-field stimulus background (LF and F) to enhance clarity.**
We have modified Figure 1 D to indicate the direction of flow field motion. We have also changed the thickness of the grey arches to indicate that the lines increased in thickness and the flow field expanded. Therefore, we modified the text of the legend of Figure 1 and relevant description of the Visual Stimulus to match figure.

**3. L 166: The rationale behind the decision to maintain both the looming stimulus and the flow field within the lower half of the dome screen is not trivial as a locust flying within a dense swarm of conspecifics encounters an extremely complex visual background also in the sky or in its entire visual field. This may thus bother the reader throughout, until it is explained only in the Discussion (paragraph starting at line 514). Consider adding a few words of explanation already at a much earlier point (Intro or Methods).**
This is a valid point. Therefore, we moved the relevant section with the discussion to the Visual Stimulus section of the Materials and Methods and modified the citation numbers.

**4. Unfortunately, the low resolution of the figures in the PDF file hampered their review.**
The low resolution may be due to the download from the server. Our original files that were submitted were high resolution.

**5. Figure 4: The figure legend is not clear enough. Please better explain the "1-3 looms" naming of the different columns. Also, what is the meaning of FF and NR?**
We have modified the legend and relevant section of the Results to clarify that 1,2, or 3 Looms refers the number of backgrounds that units responded to. FF was a typo. We have changed it to F to indicate flow field only. We have added text to the legend to indicate that NR refers to no response.

**6. Figure 6B: The peak time appears to be measured relative to the TOC, which would explain the negative values. I suggest clarifying this in the plot axes.**
We have clarified in the figure legend that peak time is relative to TOC. Placing this description on the axis title would be too long for the dimensions of the panel. We have also clarified that the peak firing rate is denoted as peak amplitude (P_a_). We also included these descriptions in the relevant sections of the Materials and Methods.

**7. 6. The " Common trends " paragraph, starting on line 424 contains many technical details that would be more appropriate in the Methods section.**
Respectfully, we disagree. The Materials and methods section describes how dimensionality reduction was performed. The paragraph that the reviewer refers to is the result of that analysis and, therefore, is more appropriate in the results section.

**8. In the Discussion section, the authors explain in length all the details of their findings, including all minor temporal aspects of the multi-unit electrophysiological response. However, what is very much missing is the behavioral significance of all this, i.e., what is the effect of a slight delay in peak amplitude, or a minor change in the decay slope of the firing rate diagram on the behavioral motor response of the locust, on its ability to escape collision or avoid predators!**

The reviewer raises an interesting point. We have added a paragraph at the end of the discussion to speculate on how optic flow effects on units and common trend responses could influence behaviour.

**Reviewer #2:**

In the manuscript ‘Background optic flow modulates responses of multiple descending interneurons to object motion in locusts’, Zhang and Gray conducted multichannel extracellular recordings from descending neurons of Locusta migratoria. Many of the recorded neurons were sensitive to a looming stimulus that mimics a sudden approach of a large object, e.g., a predator. The present study explicitly tested how the presence of optic flow cues in the background affects the neural response to a looming stimulus presented in the foreground. Optic flow cues are visual stimuli induced through self-motion and therefore omnipresent when an insect flies. In other words, testing the neural response to a looming stimulus in the presence of optic flow cues in the background represents a more naturalistic stimulus scenario and hence makes the study’s rationale highly attractive to a huge neuroscientific community. In addition to minor concerns, I have only a few major concerns.

**Unfortunately, the authors presented mostly grouped data, e.g., figure 5, which makes it difficult for the reader to evaluate the results objectively. More specifically, the reader cannot assess how the response characteristics change with different backgrounds within a unit. As all units were stimulated with all four stimuli, the strength of the author’s experimental approach is to show changes in the response profile within a unit. To appreciate the authors’ findings, it is important to present response profiles of single units in response to the four different visual stimuli.**

We have added a new supplementary figure (S1 Figure) that shows responses of units from one locust that fit into each of the 5 response categories described in Figure 5. We have also added text to the relevant section of the results to describe this figure.

**Another major point is that the recordings had been conducted in tethered, non-flying locusts. This means that the presented optic flow cues were uncoupled with the locusts’ state of locomotion (open loop). The authors must at least discuss the study’s technical drawback of recording from a stationary insect. I can imagine that a motor efference copy that closes the loop between the insect’s flight movement and the optic flow cues in the background would make the neurons more sensitive to stimuli that mismatch with the motor efference copy, i.e., a looming stimulus that approaches faster than predicted from the insect’s flight speed.**
The reviewer is correct and this is a longer term goal to further investigate the role of visual descending neurons in generation of flight escape behaviour. Currently, however, that goal was not the focus of this work and is beyond the scope of the setup we used. Nevertheless, we have added a section to the discussion to address this point.

**Major points:**
**• Line 144-174: At which velocity was the flow field (background) presented? If it was static, it is critical to call it ‘flow’. Based on the introduction, I would suspect to present a looming stimulus in the presence of optic flow that mimics a biologically plausible flight speed of the locust.**

We have renamed “circles” to “arches” to better represent their shape. We added text to the Materials and methods that describes the flow field velocity and that it represents forward motion at 3 m s-1, which is the average flight speed of a locust. We have changed the graphic representation of the flow field in Figs. 1, 3, 4, and 7 to demonstrate that the width of the arches increased during expansion.

**• Why did the authors not analyze changes in the response profile induced by different backgrounds for the remaining unit categories (category 2-5)?**
We have added a section to the results to clarify why we did not measure specific parameters form the other response categories.

**Minor points**:
**Line 52: Please also add a citation of Martin Egelhaaf’s group who studied the influence of optic flow on flight control for decades.**
We have added a citation for: 1. Egelhaaf M, Lindemann JP. Path integration and optic flow in flying insects: a review of current evidence. J Comp Physiol A. 2025;211: 375–401. doi:[10.1007/s00359-025-01734-9](https://doi.org/10.1007/s00359-025-01734-9)

**Line 64: But, see the recent work of Marion Sillies who revealed six types of direction-sensitive neurons in Drosophila. (DOI: 10.1126/sciadv.abi7112)**
We have updated this reference.

**Line 89-91: Please add a sentence about the nature of your used backgrounds. What were you presenting making them ‘naturalistic’?**
We have modified this sentence to to better represent the stimuli we presented. We also added additional text to the materials and methods to further describe the backgrounds.

**Line 90: Typo: rigidly-tethered**
We have made the change

**Line 119: Please specify how the tetrode was fabricated. Which electrodes were used to built the tetrode? Did you measure the electrode resistance before each experiment?**
We referenced the tetrode fabrication process that we followed. We have added further text to describe the tetrode impedance.

**Line 139-140: It might be helpful to add the ‘orientation °’ in Figure 1C. And please increase the size of the schematized locust in the figure.**
We have added this orientation and enlarged the size of the locust. We also added appropriate text to the figure legend.

**Line 165-167: Please explain why you decided to present the looming stimulus from below and not from above?**

Please see our response to a similar comment from Reviewer 1.

**Line 185-197: How large was the time window for spike detection? Maybe you could add a time scale in Figure 2B.**
We have added a time scale to Fig. 2B and added details to the relevant section of the text.

**Line 206: Instead of using the term 3D feature space, I prefer using terms like PCA. Especially, when you are using the abbreviation ‘PC’ in the figure which has not been introduced in the legend.**
We have changed “feature” to “principal component (PC)”.

**Line 213: ‘not nonresponded’ does not make sense**
This typo has been corrected to read “…not responded…”

**Line 218: ‘discriminated units’. Terms like ‘single units’ or ‘spike-sorted units’ are more common.**

We have changed “discriminated” to “spike sorted” throughout the document.

**Line 307: A maximal number of 23 recorded units per animal sounds a lot. Also, the mean of 16.8 units per animal is high. I wonder how the entire PCA of one animal looks. In Figure 2, you are only presenting 3 example units that were relatively easily distinguishable by eye. Is this also the case for an entire PCA?**
We have added text to the discussion to clarify the validity of the number of sorted units. For Figure 2B we have included all 20 units that were sorted from a merged file of 20 stimulus presentations to one locust. We have modified the text of the methods and caption for Figure 2 accordingly.

**Line 351-366: Category 4 (Tonic response): How does it differ from a non-responsive unit? Is the firing rate in Figure 5 for category 4 units above a background activity (of no visual stimulus)? Unfortunately, I can´t evaluate this based on the figure.**
We only categorized units that showed a statistical response to the stimulus as described in the methods. Therefore, units in category 4 were different from the non-responsive units. We have clarified in the description in the text of the firing rate trends for all categories and more correctly described the slow firing rate increase in category 4. We also added text to clarify that the categories are similar to those reported in previous studies.

**Line 376: How long does it take until the post-TOC response decays for category 5 units? In Figure 5, it is unfortunately clipped.**

The response for this category would typically decay within 500 ms after TOC. It appears to be clipped in the figure because we used the same time window as with the other categories (200 ms after TOC). We did not display data beyond this window because it is not behviourally relevant to collision avoidance. In the text of the results, we indicated that that for category 5, the firing rate increased after TOC.

**Line 385-392: Instead of writing that you found significant differences, specify your effect. For example, for the LF condition the units reached their maximal firing rate later but showed a significant shorter rise phase which reflects a more sudden change in the firing rate than in response to looming stimuli presented in absence of a flow field (LW and LG).**

This description is included in the summary paragraph at the end of this section.

**Line 399: You should specify the sample size for decay phase in the corresponding subfigure of figure 6**.
We have included the sample size in the text and figure caption.

**Line 400: Typo: ‘oof’**
We have made the correction

**Line 424-451: Please specify the rationale for the ‘common trend’ analysis. Can I interpret it as a model-based categorization of different response profiles (instead of the five categories you manually selected beforehand)?**
We added a sentence in the Dimensionality Reduction section of materials and methods as well as a reference to rationalize the dynamic factor analysis, which is further described in detail.

**Line 475: Again specify the sample size**
We have added the sample sizes to the text

**Line 522: Typo: ‘bbackground’**
We have made the correction

**Line 522-526: Justifications for the used visual stimuli must be additionally mentioned in the method section. These questions I was asking myself the entire manuscript and had to wait for the answers until the discussion**.

Please see our response to a similar comment from Reviewer 1.

**Line 532: ‘number of common trends, representing the types of responses among all responsive units’. This info must appear in the result section about the common trends.**
In response to a similar comment by Reviewer 1, we have moved a section of the discussion that described the flow field to the materials and methods. This section also included the line identified above. In the new section within the methods, we clarified that the common trends that are similar for approaches at 0° and -45° are actually from a separate dataset (originally included in a separate PhD thesis chapter) and not part of the results presented in this manuscript. This separate dataset is intended for a separate publication. The statement was made to further validate the flow field setup that required objects to approach from -45° elevation.

**Line 539: Missing punctuation mark.**
We have made the correction

**Line 585: ‘In our study’**
We have changed “my” to “our”

**Line 560-562: In line with this hypothesis, state-dependent central-complex neurons have been recently described in monarch butterflies (doi.org/10.1016/j.cub.2021.11.009).**
We have added this reference to support our suggestion.

**Figure 2:
• As you are using two different color codes (one for discriminating the signal from each electrode and one for each sorted unit) I needed some time to understand which color stands for what. I suggest keeping the raw Tetrode signal in grayscale and using the color code only for the sorted units.**

We have changed the colour of the raw recording to grey scale and further modified Figure as described in the relevant comment above. The colour code of the sorted units is the same in A and B.

**• Maybe you could rotate the PCA a little so that the distinction between the green and yellow unit becomes clearer. You could also zoom in a little.**

The new PCA has been rotated for a better view.

**• Please color code the ellipses in C so that they can be clearly distinguished from the cumulative response.**

We have changed the ellipses to dark orange for clarity and modified the text of the legend.

**• Please indicate from your 20 example units which ones were declared as responsive.**
We have identified each unit as responsive or not responsive and modified the text of the legend.

**Figure 3:
• For a better comparison across animals, I would plot on the y-axis the percentage of responsive neurons from the recorded neurons rather the absolute number.**

We have made this change

**• Please add individual data points into your boxplots.**
We have made this change

**Figure 4:
• Please explain all abbreviations used in the figure in the legend (this comment should be considered for all figures)**

We have described the stimulus abbreviations in the legend for Figure 3. We have not done this fo all figure legends as it would be redundant and the stimuli are also described in the text. Figures 3, 4, and 7 also include diagrammatic representation of the stimuli.

**• Typo: I assume, on the x-axis it should say ‘F’ instead of ‘FF’?**
We have change “FF” to “F”.

**Figure 5:
• Please indicate the number of neurons presented in each category.**
The number of neurons are reporter in S3 Table and have been added to the plots in Figure 5. We note that the original plot for Category 1 contained data from 60 units. This was a result of the graphing limitations of the software used. The revised Figure 5 contains plots from all 169 units in Category 1. Nevertheless, the general description of units in Category 1 remains unchanged.

**Figure 6:
• Please indicate the number of neurons presented in the boxplots (based on the results I suspect N = 135).**

The reviewer is correct and we have added the value of N to the figure legend.

**• Please divide the subfigure B into four subfigures (B-E). This makes it much more easier to connect the written results directly to the right subfigure. Also rearrange the boxplot figures so that they fit with the order mentioned in the results (‘peak time’, ‘rise phase’, ‘peak firing rate’, ‘decay phase’).**

We have divided the subfigures as suggested. We have left the order of the plots as is since peak time and firing rate are related parameters, as are the rise and decay phases, The text describes parameters that were significantly different or not, which is more parsimonious.

**Reviewer #3:**

The manuscript describes the analysis of electrophysiological recordings of locust 'looming detector' neurons under semi-naturalistic conditions. The animals are immersed in a type of virtual reality that allows the presentation of sky/ground simulations and a looming object in the shape of a black disc. While the looming detector has been described before, the combination with a non-uniform background is novel and worth investigating. A fairly large number of looming-responsive cells are found, many of which show peak responses in the temporal vicinity of an impending collision. The manuscript is written fairly clearly, and the conclusions seem warranted. However, I have a number of concerns regarding the statistical methods and the data availability -- in its current form, I could not reproduce the results based on the (lack of) data provided.

Major comments:

Data availability:
**While the spike detection and sorting seem pretty straightforward, the spike train analyses consitute the most interesting part of the manuscript. To replicate these analyses, the spike train time series would have to be provided -- the summary statistics in the supplements are not sufficient. Especially the more exploratory parts of the analyses, such as the categorization into five response types, are impossible to confirm without access to the spiketrains. They should be provided before the manuscript can be considered for publication.**
We have submitted the raw spike trains.

**Methods: please consult with a statisticial on the conditions under which significance tests provide meaningful results (power, test derived from hypothesis rather than post-hoc inspection of data etc.) and use them only under appropriate conditions.**
We appreciate the reviewer’s comment. We did, in fact, formulate and test specific hypotheses but neglected to list them in the introduction. We have included them, thus validating the statistical tests that we used. We also included the results a power analysis of the data in the Statistical Analysis section of the Materials and Methods.

**Details:**

**Intro:**

**line 89: "...more natural environment.." I agree that the simulated sky/ground is already more natural than previously used stimuli. But why not use the setup for the presentation of an actual sky/ground movie? The setup seems to allow for the necessary resolution etc.**
The setup we used was intentionally designed to be easily quantifiable to allow for comparisons between conditions. The stimulus generation was precisely controlled. An actual sky/ground movie would be interesting but would introduce too many confounding variables to make the analysis clear. It could be an interesting experiment in the future but is beyond the scope of the data we collected here.

**Methods:**

**line 220: 1 ms bin width, 50ms filter. Why this choice?**
This is a standard setting that we have used in previous publications and is a reasonable time window for the data we collected. It was important to use similar analysis settings to compare the findings here to previous results. There is no a priori definitive setting for the smoothing window. It is possible that detailed recordings of time constants from postsynaptic neurons could inform a strict time window setting. However, as we do not know all the postsynaptic connections form the DCMD, let alone the multiple units recorded here, doing so is unrealistic. Therefore, our setting of 50 ms is a valid and precedented window.

**line 156: time of collision (TOC). How was that determined, i.e. how large was the disc when 'collision' happened and why is that a sensible choice?**
As described in the methods, TOC was built into the code that generated object motion and accounted for the geometry of the set up. We have added a sentence in the methods to indicate that the final subtense angle of the object was 180°, which the point of collision.

**fig 2a: please put units on the time axis.**
We have added a scale bar to Fig. 2A.

**line 221: "a 2.2-second window..." in fig 2C the window seems to start 1 second before TOC, and last for a fraction of a second afterwards. Why is the rest of the response not shown?**
The time window starting 2 seconds before collision was chosen to provide a window that captured all of the neural responses, which, in practical terms began 1 second before TOC (see t_95_ in Figure 6A). For display purposes, the 1 second before TOC and 0.2 seconds after TOC provides better temporal resolution.

**fig 2C: please explain why the interval of 99% confidence (in a constant response?) is elliptic in shape. I understand why the confidence interval shrinks to size 0 at the end of the analysis interval, since then the cs(T) is equal to A (lines 228-230). But why should the confidence interval be equally small at the beginning of the interval?**
The cumulative sum and 99% confidence ellipse is a routine used by the spike train analysis software that we used (Neuroexplorer) and is an established mechanism to assess if there is a significant change in a units firing in relation to a stimulus. Two examples where it has been used previously are included as citations in the text.

**line 291: "no inherent relationship" please define what an inherent relationship is.**
We appreciate that this is not the best wording. Therefore, we changed “variables with no inherent relationship” to “Response parameters” to more accurately describe the statistical tests.

**line 308: "Multivariate Analysis of Variance (MANOVA) verified the statistical distinctiveness of these units in three-dimensional cluster space" Please describe the space in which this analysis was carried out, and what the (in)dependent variables were.**
As per the suggestion from Reviewer 2, we have clarified by changing the term “feature space” to “PC space”.

**350-366: where do the categories come from? If the data were sorted according to visual inspection, then the chi-square test is misleading, because these categories were generated from the data rather than from a hypothesis, so this constitutes 'harking'. Please remove the tests and just describe your visual impression, if that is how you arrived at the five response types.**
Please see the response to the major comment above.

**lines 379-401: please also delete the misleading significance test results and just report the descriptive statistics -- at this point there seems to be no theory/hypothesis from which a statistically testable prediction could be derived?**
Please see the response to comment above.

**line 429: "Although temporal resolution was reduced, the 50-ms-binned data captured the dynamic modulation of firing rate while significantly reducing computational complexity by a factor of
fifty." How was it determined that the 50-ms binning captures the rate modulation well?**
We have included a reference to Parkinson et al. 202, who used the same tetrode design and bin width for the PSTHS. Previous published studies in our lab consistently use a 50 ms bin width to describe the firing rate. Earlier reports described analysis with different bin widths and found that similar firing rate parameters that we describe here are well described across different values. The point being that 50 ms is an established bin width.

**line 442-443:"In response to a looming stimulus, irrespective of background types, most common trends exhibited a positive peak near the time of collision (TOC)." This seems to contradict the 5 response types depicted in fig 5. E.g. how can a constant response be explained by a positive peak near TOC? Or a valley or a peak after TOC?**
We have added text to this section of the Discussion to indicate that this finding is not surprising given the relative weight of the units with a positive peak that contributed to the common trends.

**line 492: the result of this ANOVA seem to hinge on the (arbitrary) choice of 'response start' (line 488). I recommend describing just the descriptive statistics in figure 8 and deleting the ANOVA results, which do not seem to be derived from a testable hypothesis?**
Please see the response to comment above.

**The discussion is adequate and interesting.**

We appreciate this comment
