# Supplementary material for: Background optic flow modulates responses of multiple descending interneurons to object motion in locusts
Source: PLoS One. 2025 Dec 29;20(12):e0312637. doi: 10.1371/journal.pone.0312637 (PMC12747386; doi:10.1371/journal.pone.0312637)
Supplement: Attachment — Submitted filename: Response to reviewers comments_2nd review.docx [file pone.0312637.s008.docx]

We thank the reviewers for their carefully considered and constructive comments on the manuscript. We have made an effort to incorporate all suggestions and edits and provide a response to each comment below in red text.

Reviewer #1: All my comments have been adequately addressed, and the paper may be accepted for publication in its current state

We thank the reviewer for their positive feedback and support for our manuscript.

Reviewer #2: The manuscript has substantially improved after the revision. My last major concern is about the spike-sorting approach which gets exemplified in figure 2. I appreciate the authors’ transparency by showing all 20 clusters in the PCA but unfortunately, I can`t distinguish the central clusters that are entirely overlapping in the current figure, e.g. magenta, white and yellow cluster. I strongly recommend showing the central clusters in a magnified view, so that the reader can assess the sorting.

We have modified Fig 2b to show all unit clusters and enlarged central clusters.

Similar problems with quality assessment of spike sorting appear in the spike shapes presented on top of B. Too many clusters are overlapping, making it impossible to see every cluster’s spike shape. In addition to this, colors are replicating, i.e., three clusters are shown in red, three in yellow … . This makes it impossible to assign each cluster to the corresponding spike shape. I therefore strongly recommend splitting the spike-shape images into multiple subimages.

To address the reviewer's valid concerns, we have reorganized Figure 2. We added a new panel dedicated to the spike shapes of the central clusters, using a distinct and non-repeating color palette to ensure each waveform is clearly attributable to its cluster. We found this approach provides a more readable and effective visualization than a grid-style layout of all 20 units.

Figure 2: Please increase the size of Fig. 2A, the sorted units (especially the small amplitude ones) are barely visible.
We have enlarged the bottom section of Fig 2a. We have also changed the background of the bottom section from black to white, making the colour-coded units easier to see.

Line 63: After the revision, the term ‘similar to mice’ does not fit anymore as you were previously referring to four types of direction selective neurons in mice
Thank you for pointing this out. We have removed the phrase.

Line 132: Please give some specs about the silver ground wire (diameter, fabricate)
Thank you for pointing this out. We have added the required information.

Line 148: To me, the arrows appear yellow
That is a valid point. We have updated the figure legend.

Line 248: What exactly did you statistically compare with the MANOVA? The distance between the clusters in the PCA, e.g., the centroids or the spike shapes between clusters? Please give a few more infos on that.
We used the built-in MANOVA test provided by the Offline Sorter software to statistically validate the separation of our sorted units. This test compares the distributions of spike data points in the 3D feature space, defined by the first three principal components. The independent variable for the test is the assigned unit (cluster), and the dependent variables are the feature vectors (i.e., the PC scores) for each individual waveform within those units. These information have been added to the text too.

Fig 3: “N” for data points is missing in the legend

Thank you for pointing this out. We have updated the info.

Line 390: Please specify individual data points (individual locusts?)

Yes. Individual data points represent different animals. We have updated the info in the figure legend.

Line 434-435: Please place this info to line 421

Thanks for the comment. We have made the change.

Category 5: To me it looks like that the firing rate first drops before TOC and increases afterwards

This is a valid point. However, this drop was also observed in Categories 2, 3, and 4. The distinct feature of Category 5 is the prominent increase after TOC, which resembles some common trends from the DFA (CT5 for LW, CT7 for LG, and CT4 for LF).

Figure 6: Please highlight the median of the boxplots in red (especially for the LF condition it is difficult to see the median)

We have updated figures 3, 6 and 8, highlighting the median line in red. The figure legends have been updated as well.

Update: The previous Fig 6 and 8 are now Fig 7 and 9, respectively, since the previous Fig S1 was moved to Fig 6.

Line 489-503: Please place this passage before line 448. It is more intuitive to read first about the different categories and then getting the explanation why you were focusing on category 1 for detailed analysis.

This is a good point. We have modified the order of corresponding paragraphs to make the flow smoother.

Line 492-493: Do you suspect all units to be putative DCMD or only category 1 units? This is still unclear as it is written here.
Thanks for pointing it out, and the description can indeed cause confusion. Therefore, we have updated the text from “these categories” to “category 1”, since we were only talking about category 1 here.

Figure S1: Please place this figure into the main paper. It is the only figure that presents raw data of the effect of the background on the looming response. Please also enlarge the region around TOC so that the reviewer can better see the effect for category 1.

We appreciate this valuable suggestion. We have moved the figure into the main paper as the new Fig 6. Furthermore, to make the effect for category 1 easier to see, we have added an inset panel that shows a magnified view of the response around the time of collision (TOC).

Line 523: Sometimes you write “fig” and sometimes you write “figures”. Please stay consistent.
We have modified the corresponding texts to ensure consistency.

Figure 7 legend: To get it right: These PSTHs have a binsize of 50 ms? If this is the case please add this info in the legend.

We have added the information in the figure legend.

Update: The previous Fig 7 is now Fig 8, since the previous Fig S1 was moved to Fig 6.

Line 543: What does the color gradient in the heatmap represents?

The heatmap represents the factor loadings of each unit to the specific common trend, i.e., the correlation of each unit to the common trend. We have added further clarification in the Fig 7 figure legend.

Update: The previous Fig 7 is now Fig 8, since the previous Fig S1 was moved to Fig 6.

Is there a reason why you did not compare t95 in figure 6?
Thanks for pointing this out. Initially, we just compared the peak time, rise phase, and decay phase, as routinely done in previous studies. The rise phase was the period between t95 and peak, and we found significant differences in peak time and rise phase already. Since a delayed peak and shortened rise phase would naturally indicate a delayed t95, we did not compare t95. However, since we did compare t95 later in Fig 8, we agree that it is better to include t95 comparison in Fig 6 as well. Therefore, we have included the One-way RM ANOVA comparison of t95 in the corresponding results section, and added a section F in Fig 6 to show the result.

Update: The previous Fig 6 and 8 are now Fig 7 and 9, respectively, since the previous Fig S1 was moved to Fig 6.

Line 576: Similar to figure 6? Not 5

That is correct. Thanks for pointing it out. We have made this edit.

Update: The previous Fig 6 is now Fig 7, since the previous Fig S1 was moved to Fig 6.

Line 623: Also bumblebees estimate flight height by using optic flow (10.1242/jeb.249763). Please add this reference to your discussion

Thanks for suggesting the reference. We have added it to the discussion.

Reviewer #3: Thank you for providing the data for result replication and additional analyses, I expect this will be useful for the scientific community. I assume the link to the data will be added to the final manuscript, too?

Thanks. We will include the link in the final manuscript.

Regarding the clustering analysis: I still strongly recommend removing the misleading chi-square tests, since (line 419ff) "We identified similar categories based on the trends of firing rate changes over time in response to a similar stimulus ..." i.e. the categories were defined by "eyeballing" the data, which makes significance tests un-applicable and constitutes 'harking'. If these clusters were devised by applying a statistical model to the data (e.g. Gaussian mixtures or similar), then a bayesian model evidence or BIC score could be used to identify the number of clusters.

We have removed the chi-squared test, and only kept the description of the distribution of units between different backgrounds.
