# Supplementary material for: Background optic flow modulates responses of multiple descending interneurons to object motion in locusts
Source: PLoS One. 2025 Dec 29;20(12):e0312637. doi: 10.1371/journal.pone.0312637 (PMC12747386; doi:10.1371/journal.pone.0312637)
Supplement: Attachment — Submitted filename: Responses to reviewers comments_3rd review.docx [file pone.0312637.s009.docx]

We thank the reviewers for their carefully considered and constructive comments on the manuscript. We have made an effort to incorporate all suggestions and edits and provide a response to each comment below in red text.

Reviewer #2: Congratulations to the authors who did a good job in revising the manuscript. Thank you for addressing all my concerns and accepting my suggestions. From my side, the manuscript is ready for publication.

We thank the reviewer for their positive feedback and support for our manuscript.

Reviewer #4: This is a strong and carefully executed study. The authors examine how background optic flow, simulating self motion, influences neural processing of looming stimuli in locusts. Multi-channel electrophysiology shows that optic flow does not change how many neurons respond, but it shifts response timing later (for both onset and peak) and results in fewer common population trends. The analysis is solid and the work provides valuable insight into how collision detection operates in visually complex conditions. The manuscript is nearly ready for publication and would benefit from the following clarifications and contextual additions.

A. Suggested theoretical context

To strengthen the link between the neurophysiology and real-world behavior, the discussion could acknowledge recent work showing how vision guides collision avoidance and collective movement in locusts. Your results provide neural grounding for models that treat visual motion cues as drivers of group coordination and selective responses in crowded environments.

Consider citing the following works near line 690 or in the final discussion paragraph:

Bleichman I, Yitzhaki O, Bernat NM, et al. 2024. The visual stimuli attributes instrumental for collective-motion tuning in desert locusts. PNAS Nexus. https://doi.org/10.1093/pnasnexus/pgae537

Krongauz DL, Lazebnik T. 2023. Collective evolution learning model for vision based collective motion with collision avoidance. PLOS ONE. https://doi.org/10.1371/journal.pone.0270318

Egelhaaf M, et al. 2023. Optic flow based spatial vision in insects. J Comp Physiol A. https://doi.org/10.1007/s00359-022-01645-z

These papers directly connect visual motion processing to group coordination, collision avoidance and naturalistic movement environments.

We appreciate this suggestion to strengthen the ecological context of our findings. We have added the suggested text and citations to the final paragraph of the Discussion. We explicitly link our results to models that treat visual motion cues as drivers of group coordination and collision avoidance in crowded environments.

B. Clarification in the discussion

In the concluding paragraph, the authors suggest that delayed neural responses in the presence of optic flow may slow behavior or fine tune responses. This can be clarified as a likely adaptive trade-off. When optic flow signals self motion, the nervous system may increase its decision threshold to prevent unnecessary avoidance actions. At the same time, once the threshold is crossed, the reduced variability of population responses suggests a more stable and decisive motor command. Briefly stating this balance between avoiding false alarms and maintaining reliable escape behavior will help tie neural timing and population coding to ecological context.

Suggested phrasing idea:
In conditions containing self-generated optic flow, the system may adopt a more conservative triggering policy, delaying responses to avoid false alarms, while producing a more consistent output once activation occurs.

We agree that framing this as an adaptive trade-off improves the interpretation. We have revised the concluding paragraph of the Discussion to clarify that in conditions containing self-generated optic flow, the system likely adopts a more conservative triggering policy—delaying responses to prevent false alarms while maintaining consistent output once activation occurs.

C. Minor clarity suggestion in Results

In the common trends results, two key findings are presented for the flow condition: fewer common trends and delayed rise time. To improve clarity, consider adding one sentence in the Results or early in the Discussion stating that this indicates a more stereotyped and temporally shifted population code under optic flow. This will help readers follow the logical step from analysis to behavioral interpretation.

We have added a sentence to the "Common Trends" section of the Results to clarify this point. The text now explicitly states that the reduction to 5 common trends indicates a more stereotyped and temporally shifted population code in the presence of optic flow.
